# Low-Complexity Joint 3D Super-Resolution Estimation of Range Velocity and Angle of Multi-Targets Based on FMCW Radar

**DOI:** 10.3390/s22176474

**Published:** 2022-08-28

**Authors:** Yingchun Li, Qi Long, Zhongjie Wu, Zhiquan Zhou

**Affiliations:** 1 School of Information Science and Engineering, Harbin Institute of Technology, Weihai 264209, China; 2 School of Electronic Science, National University of Defense Technology, Changsha 410073, China

**Keywords:** FMCW radar, super resolution, array signal processing

## Abstract

Multi-dimensional parameters joint estimation of multi-targets is introduced to implement super-resolution sensing in range, velocity, azimuth angle, and elevation angle for frequency-modulated continuous waveform (FMCW) radar systems. In this paper, a low complexity joint 3D super-resolution estimation of range, velocity, and angle of multi-targets is proposed for an FMCW radar with a uniform linear array. The proposed method firstly constructs the size-reduced 3D matrix in the frequency domain for the system model of an FMCW radar system. Secondly, the size-reduced 3D matrix is established, and low complexity three-level cascaded 1D spectrum estimation implemented by applying the Lagrange multiplier method is developed. Finally, the low complexity joint 3D super-resolution algorithms are validated by numerical experiments and with a 77 GHz FMCW radar built by Texas Instruments, with the proposed algorithm achieving significant estimation performance compared to conventional algorithms.

## 1. Introduction

In recent years, with the development of millimeter-wave (mm-wave) semiconductor technology, the application of frequency modulated continuous wave (FMCW) radar sensor systems has become easy and popular [1] and many commercial radars can be used for automotive applications [2,3], UAV detection [4,5], and medical diagnosis [6,7,8], etc. Radar sensor systems play an important role in these applications to obtain state features such as range, velocity, and angle of targets. Therefore, various estimation algorithms based on FMCW radar have also been proposed due to the wide application of FMCW radar sensor systems. In those traditional algorithms [9,10,11], the range, radial velocity, and angle information of the targets can be estimated by taking the 3D fast Fourier transform (FFT) on the beat-frequency (BF) signal, which is achieved by the dechirping operation of the received and transmitted signal. Although this algorithm is simple and efficient to use, its resolution is limited by hardware aspects such as the bandwidth of the transmitted signal, the area of the receiving antenna aperture and the number of chirps transmitted [12]. The resolution of the radar system often cannot meet our actual needs due to physical hardware limitations, which has led to the super-resolution estimation algorithm based on FMCW radar being intensively researched, especially for multi-targets detection scenarios.

The conventional one-dimensional (1D) multiple signal classification (MUSIC) algorithm [13,14], Root-MUSIC algorithm [15,16], and ESPRIT algorithm [17,18], etc., provide super-resolution estimation through the subspace-based technique. However, these algorithms only estimate the direction of arrival (DOA) and the estimation of only one parameter cannot meet our actual needs. Moreover, the estimation of range, velocity, and angle can be processed sequentially, solving each parameter estimation problem independently, but this leads to a severe pairing problem of the estimated results as the number of targets increases [19]. Therefore, many joint estimation algorithms without pairing have been suggested in recent years. For example, the L-shaped, matrix or other shapes of receiving antenna array are used to implement the algorithm for joint super-resolution estimation of elevation and azimuth based on the two-dimensional (2D) MUSIC algorithm [20,21]. Additionally, the 2D MUSIC algorithm [22,23,24] achieves super-resolution joint estimation of range and angle by extending conventional 1D MUSIC for the angle domain to 2D MUSIC for the range-angle domain. However, the difficulty of this kind of algorithm in practice is that a lot of calculations are required in the process of constructing the covariance matrix and multi-dimensional peak searching; especially when the dimension is higher, the calculation amount is huge. To reduce the amount of calculation, the methods of dimensionality reduction and beam projection are applied to the 2D MUSIC algorithm [25,26,27] and the ESPRIT algorithm is used to replace the peak search process of MUSIC [28] in 2D. However, such low-complexity and super-resolution estimation algorithms are rarely studied in the joint 3D estimation of range, velocity, and angle. Although some 3D joint estimation algorithms based on 3D MUSIC have also been proposed [29,30], they have not solved the problem of high complexity in 3D.

Hence, in this paper, we propose a low-complexity super-resolution joint 3D estimation algorithm of range velocity and angle of multi-targets. Based on 3D MUSIC, this algorithm reduces the amount of computation through two-part processing. First, we reduce the amount of matrix computation and data storage by extracting useful frequency information in the beat signal. Then, the conventional 3D peak searching is transformed into three-level cascaded 1D searching by applying the Lagrange multiplier method. Additionally, this algorithm uses a three-dimensional spatial smoothing technique [31] to solve the problem of coherent echoes. The results of simulation and actual experiments show that the algorithm not only substantially reduces computation, but also maintains super-resolution ability.

This paper is organized as follows: Section 2 presents the radar system and signal model. Section 3 introduces this low-complexity super-resolution algorithm. The results of experiments and the performance analysis of this algorithm are discussed in Section 4. Finally, the conclusion is presented in Section 5.

## 2. Signal Model

**Figure 1:**
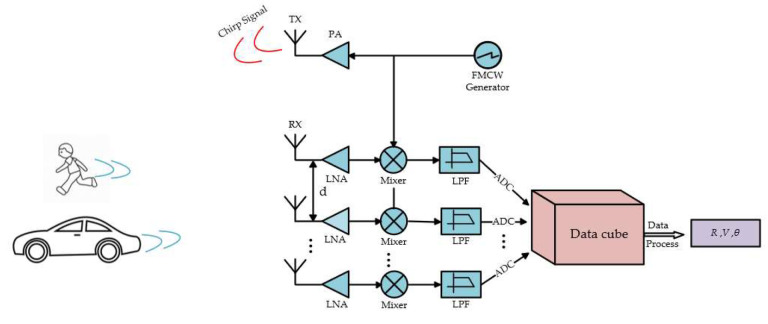
Block diagram of FMCW radar.

Consider a SIMO FMCW radar system, which consists of a uniform linear array (ULA), and the system block diagram is as shown in Figure 1:

A chirp signal consists in transmitting a frequency-modulated signal which exhibits a linear frequency increase or decrease over the bandwidth of *B_w_* Hz in the duration time of *T_c_* s. The linear frequency increase version transmitted by the FMCW radar can be modeled as [32]:

(1) $$s_{t}\left(t\right)=\left\{\begin{array}{lll} e^{j2\pi \left(f_{c}t+\frac{\mu }{2}t^{2}\right)} & & 0\leq t<T_{c}\\ 0 & & \;\mathrm{elsewhere} \end{array}\right.$$

where *f_c_* is the carrier frequency, *μ = B_w_/T_c_* is the ratio of the bandwidth of the transmitted chirp signal to its duration time of sweep. Considering *K* narrowband sources impinging on the ULA of the radar system reflected by *K* moving targets in the far field, the received signals can be defined as:

(2) $$\begin{array}{l} s_{r}\left(t\right)=\sum_{k=1}^{K}s_{t}\left(t-\tau_{k}\right)+w\left(t\right)\\ \;\;\;\;\;\;\;\;\;\;\approx \sum_{k=1}^{K}e^{j2\pi \left[\frac{\mu }{2}{\left(t-\frac{2R_{k}}{c}\right)}^{2}\right]}e^{j2\pi \left[f_{c}\left(t-\tau_{k}\right)\right]}+w\left(t\right) \end{array}$$

(3) $$\tau_{k}=\frac{1}{c}\left\{2\left[R_{k}+\left(l-1\right)T_{c}V_{k}+V_{k}t\right]+\left(n-1\right)d\sin\theta_{k}\right\}$$

where *K* is the number of targets, *R_k_*, *θ_k_*, *V_k_* are range, angle, and velocity of the *k*-th target, respectively, *c* is the speed of light, and *d* is the spacing of the uniform linear array antenna; *n =* 1*…N*, *N* is the number of elements of the received antenna array, *l =* 1*…L*, *L* is number of transmitted chirps, *w*(*t*) is the transformed additive white Gaussian noise (AWGN) signal. The item *τ*_k_ stands for the phase shift in the received chirp signal, and it is induced by range, velocity, and angle at the same time, as explained in [33]. To be more specific, the item *R_k_* + (*l* − 1)*T_c_V_k_* + *V_k_t* denotes the instant range at time *t* for the received *l*-th chirp reflected from the *k*-th moving target, and it deduces the phase shift induced by range and velocity. Meanwhile, the item (*n* − 1) sin*θ_k_* denotes the angle for the *n*-th sensor element of the incoming signal reflected from the *k*-th target, and it deduces the phase shift induced by angle. The beat signals can be achieved from the received signals through mixers, and the down-converted signal can be expressed as:

(4) $$\begin{array}{l} s_{b}\left(t\right)=s_{r}\left(t\right)s_{t}\left(t\right)\\ \;\;\;\;\;\;\;\;\;=\sum_{k=1}^{K}b_{k}e^{j2\pi \frac{2\mu }{c}\left(R_{k}+\frac{f_{c}}{\mu }V_{k}\right)t}e^{j2\pi \frac{f_{c}}{c}\left(l-1\right)T_{c}V_{k}}e^{j2\pi \frac{f_{c}}{c}\left(n-1\right)d\sin\theta_{k}}+w\left(t\right) \end{array}$$

where *b_k_* is the complex amplitude of the *k*-th received signal reflected by the *k*-th target, after analog-to-digital conversion, we can get the discrete beat signal:

(5) $$S_{b}\left[n,m,l\right]=\sum_{k=1}^{K}b_{k}e^{j2\pi \frac{2\mu }{c}\left(R_{k}+\frac{f_{c}}{\mu }V_{k}\right)\frac{m-1}{f_{s}}}e^{j2\pi \frac{f_{c}}{c}d\left(n-1\right)\sin\theta_{k}}e^{j2\pi \frac{f_{c}}{c}2\left(l-1\right)T_{c}V_{k}}+w\left[n,m,l\right]$$

where *f_s_* is the sampling frequency, *m =* 1*…M*, *M* is the number of time samples of one chirp signal, and *w*[*n*, *m*, *l*] is the AWGN signal after discretization.

**Figure 2:**
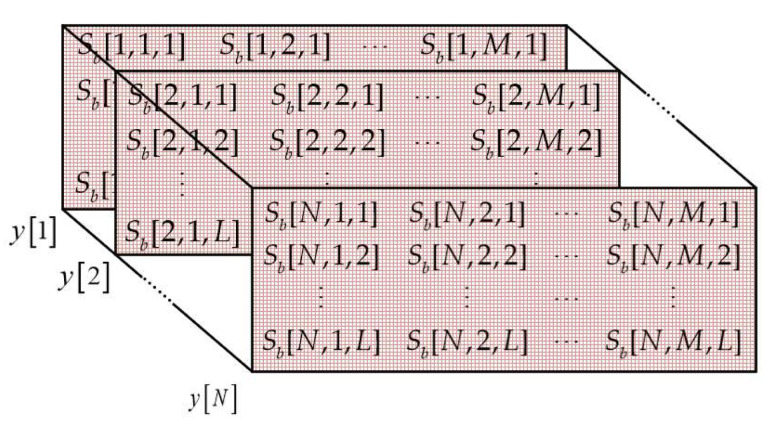
The received radar data cube.

The received radar data cube with dimension *N × M × L* can be constructed as shown in Figure 2. In the Figure 2, *y*[*n*] is the 2D data matrix of beat signal received by the *n*-th antenna and can be described as Equation (6), *W* is additive white Gaussian noise matrix.

(6) $$y\left[n\right]=\left[\begin{array}{cccc} S_{b}\left[n,1,1\right] & S_{b}\left[n,2,1\right] & \cdots & S_{b}\left[n,M,1\right]\\ S_{b}\left[n,1,2\right] & S_{b}\left[n,2,2\right] & \cdots & S_{b}\left[n,M,2\right]\\ \vdots & \vdots & \cdots & \vdots \\ S_{b}\left[n,1,L\right] & S_{b}\left[n,2,L\right] & \cdots & S_{b}\left[n,M,L\right] \end{array}\right]+W\in {\mathbb{C}}^{L\times M}$$

## 3. The Proposed Low-Complexity Super-Resolution Algorithm

In the previous multi-dimensional joint estimation [22,23,24], matrix operations and multi-dimensional peak search are the main reasons for the high complexity. For coherent signals, smoothing is necessary [34]. In this paper, matrix block selection and smoothing are firstly performed, and then the algorithm to reduce the complexity is tried.

### 3.1. Targets-Located Blocks Selection

In Section 2, the time dimension of the 3D radar data cube is much larger than the angle and velocity dimensions. Selecting the effective time dimension blocks, which contain the range information of targets, can significantly reduce the size of the conventional 3D covariance matrix.

**Figure 3:**
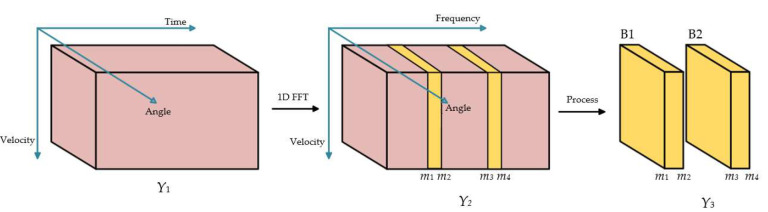
Frequency domain processing.

As shown in Figure 3, the target-located blocks of interest corresponding to temporal frequencies can be selected from the matrix Y_2_, which can be achieved by performing 1D FFT in range domain (range-FFT). Those peaks in temporal frequencies will locate in several blocks, for example: Block 1(B1) with index range [*m*_1_, *m*_2_] and Block 2(B2) with index range [*m*_3_, *m*_4_], and then the two blocks of data are jointly estimated, respectively. The relationship between range and temporal frequency is: *R = f_c_*/2*μ*. This process has two other advantages: one can roughly estimate the approximate range, and the other can filter part of the white noise to improve the signal-to-noise ratios (SNR). The targets-located blocks selection process is as shown in Figure 3.

### 3.2. Decorrelation Processing

**Figure 4:**
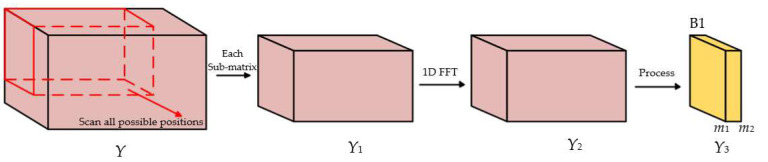
Frequency domain processing and spatial smoothing.

The eigenvalue decomposition (EVD) of the covariance matrix can obtain the signal subspace and the noise subspace. When the target echo signals are correlated, the size of the signal subspace will no longer be equal to the number of targets [35,36]. It is necessary to decorrelate the data, and the use of spatial smoothing technology is an effective means to decorrelate related signals. Frequency domain processing and spatial smoothing need to be done together. The process is as shown in Figure 4.

We define a small cube of size [*h*_1_ × *h*_2_ × *h*_3_], which is identified with the red frame in Figure 4, and scan all possible positions in the radar data cube, there are then *p*_1_ *= N* − *h*_1_ *+* 1 positions in the angle dimension, *p*_2_ *= M* − *h*_2_ *+* 1 positions in the time dimension, and *p*_3_ *= L* − *h*_3_ *+* 1 positions in the velocity dimension, and each sub-matrix needs to be processed on the frequency domain as in Figure 3. The phase shift between the adjacent samples in *h*_1_ dimension, *h*_2_ dimension, and *h*_3_ dimension are induced by angle, range, and velocity, respectively, as shown in [36].

Perform frequency domain processing on the data of each sub-matrix [*h*_1_ × *h*_2_ × *h*_3_], and then get the covariance matrix of each block. This paper takes the selected block B1 which contains $\hat{K}$ targets as an example, and denotes its data matrix as *Y*_3_ for simplicity. *Y*_3_ is the sub-matrix with range index [*m*_1_, *m*_2_] of *Y*_2_, and *Y*_2_ is the matrix of *Y*_1_ after range-FFT, and *Y*_1_ can be expressed as:

(7) $$Y_{1}=Y\left[{\tilde{p}}_{1}:\left({\tilde{p}}_{1}+h_{1}-1\right),{\tilde{p}}_{2}:\left({\tilde{p}}_{2}+h_{2}-1\right),{\tilde{p}}_{3}:\left({\tilde{p}}_{3}+h_{3}-1\right)\right]$$

**Figure 5:**
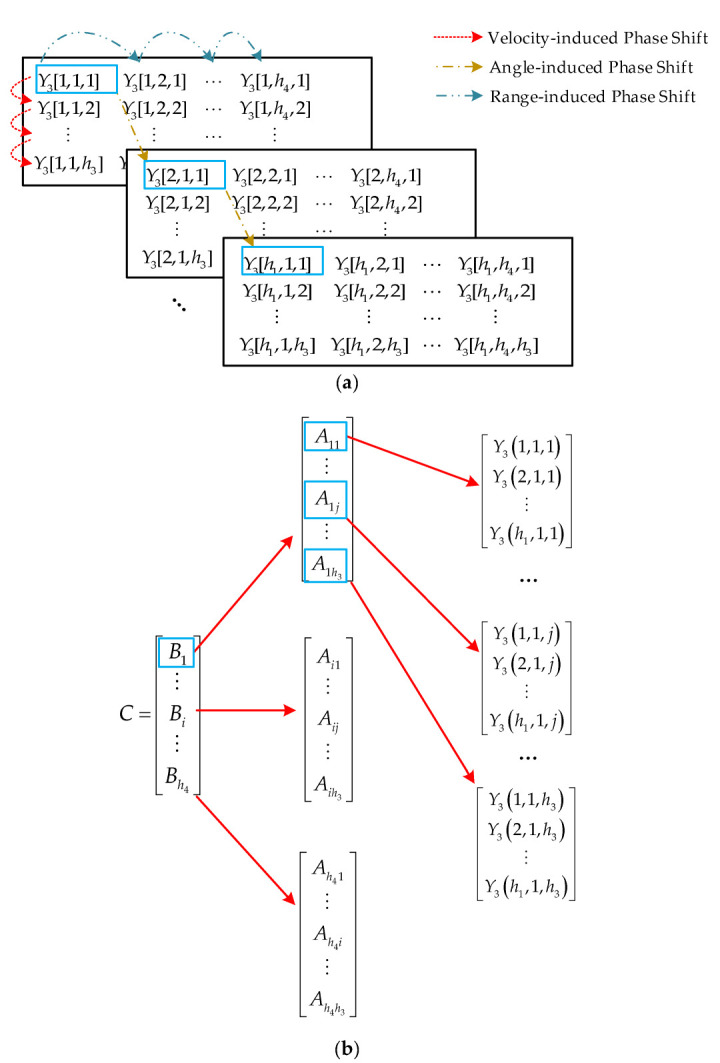
Understanding of the 3D steering vector *C*, (**a**) three kinds of phase shifts, (**b**) constructions of vector elements.

Three exponential items, which respectively contain the phase shift induced by range, angle, and velocity, are presented in Equation (5). The conceptual understanding of three kinds of phase shift can be found in Figure 5a, and each kind of phase shift has its corresponding 1D steering vector for conventional 1D MUSIC. It is important to notice that the exploitation of orthogonality between signal subspace and noise subspace remains true for the extension to 3D MUSIC. The phase shift in the received signal is obviously comprised by the three kinds of phase shifts according to Equation (5), and thus the steering vector for the 3D MUSIC is the Kronecker product of three above-mentioned 1D steering vectors. We call the steering vector for the 3D MUSIC as 3D steering vector, which contains three kinds of 1D steering vectors, and the search space for 3D MUSIC is a 3D grid of range, velocity, and angle. Therefore, the covariance matrix of B1 can be constructed as:

(8) $$D=\frac{1}{p_{1}p_{2}p_{3}}\sum_{{\tilde{p}}_{1}=1}^{p_{1}}\sum_{{\tilde{p}}_{2}=1}^{p_{2}}\sum_{{\tilde{p}}_{3}=1}^{p_{3}}\left(\frac{1}{h_{1}h_{4}h_{3}}CC^{H}\right)$$

where *h*_4_ = *m*_2_ − *m*_1_ + 1, *C* = *vec*(*Y*_3_) is the 3D steering vector, and the operation *vec*() denotes vectorization of a matrix contained within, namely, reshaping the matrix with size *h*_1_ × *h*_4_ × *h*_3_ to a vector with size *h*_1_*h*_4_*h*_3_ × 1. To be more specific, if we define vector *A_ij_* =*Y*_3_(:,*i*,*j*) with *i* = 1,2,…., *h*_4_ and *j* = 1,2,…., *h*_3_, the size of vector *A_ij_* is *h*_1_ × 1. Then, we define vector *B_i_* = [*A_i_*_1_, *A_i_*_2_, …, *A_ih_*_3,_] with size *h*_1_*h*_3_ × 1, and the interested 3D steering vector ***C*** can be denoted as ***C*** = [*B_i_*, *B_i_*,…, *B_h_*_4_] with size *h*_1_*h*_4_*h*_3_×1. Obviously, *A_ij_* contains the phase shift induced by angle, *B_j_* contains the phase shift induced by angle and velocity, and ***C*** contains the phase shift induced by angle, velocity, and range at the same time. The construction of 3D steering vector ***C*** is as shown in Figure 5b.

### 3.3. Low Complexity Joint 3D Estimation of Range-Velocity-Angle

The signal subspace and the noise subspace can be obtained after performing EVD on the constructed covariance matrix *D*:

(9) $$D=U\Lambda U^{H}$$

where $\Lambda =\left[\lambda_{1},\lambda_{2},\cdots ,\lambda_{h_{1}h_{4}h_{3}}\right]$, the noise subspace can be defined as:

(10) $$U_{N}={\left[U\left(\hat{K}+1\right),U\left(\hat{K}+2\right),\cdots ,U\left(h_{1}h_{4}h_{3}\right)\right]}_{h_{1}h_{4}h_{3}\times \left(h_{1}h_{4}h_{3}-\hat{K}\right)}$$

Defining the first phase item *R* + *f_c_V*/*μ* of Equation (5) as *G*, the 3D MUSIC spectrum can be calculated as:

(11) $$P\left(G,\theta ,V\right)=\frac{1}{{\left[\beta \left(G\right)⊗\upsilon \left(V\right)⊗\alpha \left(\theta \right)\right]}^{H}U_{N}U_{N}^{H}\left[\beta \left(G\right)⊗\upsilon \left(V\right)⊗\alpha \left(\theta \right)\right]}$$

where

$$\alpha \left(\theta \right)={\left[1,e^{j2\pi \frac{f_{c}}{c}d\sin\theta },\cdots ,e^{j2\pi \frac{f_{c}}{c}d\left(h_{1}-1\right)\sin\theta }\right]}_{1\times h_{1}}^{Τ}\;\upsilon \left(V\right)={\left[1,e^{j2\pi \frac{f_{c}}{c}2T_{c}V},\cdots ,e^{j2\pi \frac{f_{c}}{c}2(h_{3}-1)T_{c}V}\right]}_{1\times h_{3}}^{Τ}\;\tilde{\beta }\left(G\right)={\left[1,e^{j2\pi \frac{2S}{c}G\frac{1}{f_{s}}},\cdots ,e^{j2\pi \frac{2S}{c}G\frac{h_{2}-1}{f_{s}}}\right]}_{1\times h_{2}}^{Τ}$$

the *β*(*G*) is index range [*m*_1_, *m*_2_] of $\tilde{\beta }(G)$ after FFT, ⊗ denotes Kronecker product. The item *β*(*G*) ⊗ *υ*(*V*) ⊗ *α*(*θ*) usually called the 3D steering vector, which represents the set of phase-delays for the received signal at each sensor element. Since three arguments specify the testing vector, the calculated spectrum *P*(*G*, *θ*, *V*) is a 3D matrix and the estimations of range, velocity, and angle of all targets can be achieved from the value of 3D peaks of it. 

For the calculated 3D MUSIC spectrum *P*(*G*, *θ*, *V*), conventional joint estimation methods find the $\hat{K}$ maximum values by 3D peak searching, and the range, velocity and angle of multi-targets can be estimated by the indexes of the corresponding 3D peak. However, the 3D peak searching presents a heavy computational burden. The conventional Lagrange multiplier method [25,26] is adopted to reduce the computational complexity of 3D peak searching, so we define:

(12) $$\begin{array}{l} M\left(G,V,\theta \right)=\frac{1}{P\left(G,V,\theta \right)}={\left[\beta \left(G\right)⊗\upsilon \left(V\right)⊗\alpha \left(\theta \right)\right]}^{H}U_{N}U_{N}^{H}\left[\beta \left(G\right)⊗\upsilon \left(V\right)⊗\alpha \left(\theta \right)\right]\\ \;\;\;\;\;\;\;\;\;\;\;\;\;\;\;\;\;\;\;\;\;\;\;\;\;\;\;\;\;\;\;\;\;\;\;\;\;\;={\left[\upsilon \left(V\right)⊗\alpha \left(\theta \right)\right]}^{H}Q_{1}\left(G\right)\left[\upsilon \left(V\right)⊗\alpha \left(\theta \right)\right] \end{array}$$

where $Q_{1}\left(G\right)={\left[\beta \left(G\right)⊗{Ι}_{h_{1}h_{3}}\right]}^{H}U_{N}U_{N}^{H}\left[\beta \left(G\right)⊗{Ι}_{h_{1}h_{3}}\right]$. Then, the above problem becomes a quadratic optimization problem, and the Lagrange multiplier method is used to reduce its dimension. We consider using $e_{1}^{H}\left[\upsilon \left(V\right)⊗\alpha \left(\theta \right)\right]=1$,$e_{2}^{H}\alpha \left(\theta \right)=1$ to eliminate the trivial solution of zero, where $e_{1}={\left[1,0,\ldots 0\right]}^{Τ}\in {\mathbb{R}}^{h_{1}h_{3}\times 1}$,$e_{2}={\left[1,0,\cdots 0\right]}^{Τ}\in {\mathbb{R}}^{h_{1}\times 1}$. This optimization problem can be defined as:

(13) $$\begin{array}{l} \underset{G,V,\theta }{\min}{\left[\upsilon \left(V\right)⊗\alpha \left(\theta \right)\right]}^{H}Q_{1}\left(G\right)\left[\upsilon \left(V\right)⊗\alpha \left(\theta \right)\right]\\ s.t.\;e_{1}^{H}\left[\upsilon \left(V\right)⊗\alpha \left(\theta \right)\right]=1,e_{2}^{H}\alpha \left(\theta \right)=1 \end{array}$$

Let $\upsilon \left(V\right)⊗\alpha \left(\theta \right)=T\left(V,\theta \right)$, the cost function, be:

(14) $$L\left(G,V,\theta \right)=T{\left(V,\theta \right)}^{H}Q_{1}\left(G\right)T\left(V,\theta \right)-\lambda \left(e_{1}^{H}T\left(V,\theta \right)-1\right)-\eta \left(e_{2}^{H}\alpha \left(\theta \right)-1\right)$$

where *λ* and *η* are constant. Take the derivative of *L*(*G*, *θ*, *V*) as:

(15) $$\frac{\partial }{\partial T\left(V,\theta \right)}L(G,V,\theta )=2Q_{1}\left(G\right)T\left(V,\theta \right)+\lambda e_{1}=0$$

(16) $$\frac{\partial }{\partial \alpha \left(\theta \right)}L\left(G,V,\theta \right)=2Q_{2}\left(G,V\right)\alpha \left(\theta \right)+\eta e_{2}=0$$

where $Q_{2}\left(G,V\right)={\left[\beta \left(G\right)⊗\upsilon \left(V\right)⊗I_{h_{1}}\right]}^{H}U_{N}U_{N}^{H}\left[\beta \left(G\right)⊗\upsilon \left(V\right)⊗I_{h_{1}}\right]$

Firstly, according to Equation (15), we can get $T\left(V,\theta \right)=\mu Q_{1}^{-1}\left(G\right)e_{1}$, where *μ* is a constant. For $e_{1}^{H}T\left(V,\theta \right)=1$, $\mu =\frac{1}{e_{1}^{H}Q_{1}{\left(G\right)}^{-1}e_{1}}$, and to get $\hat{T}\left(V,\theta \right)=\frac{Q_{1}{\left(G\right)}^{-1}e_{1}}{e_{1}^{H}Q_{1}{\left(G\right)}^{-1}e_{1}}$. Taking $\hat{T}\left(V,\theta \right)$ into Equation (13), *G* can be estimated as:

(17) $$\begin{array}{l} \hat{G}=\underset{G}{\min}\frac{1}{e_{1}^{H}Q_{1}{\left(G\right)}^{-1}e_{1}}\\ \;\;\;=\underset{G}{\max}e_{1}^{H}Q_{1}{\left(G\right)}^{-1}e_{1} \end{array}$$

Therefore, through a 1D local search at $G\subseteq \left(\frac{m_{1}c}{2\mu },\frac{m_{2}c}{2\mu }\right)$, the ${\hat{G}}_{k}\left(k=1,\cdots ,\hat{K}\right)$ of the *k*-th target can be obtained.

Then, the Equation (16) can be rewritten as:

(18) $$\frac{\partial }{\partial \alpha \left(\theta \right)}L\left({\hat{G}}_{k},V,\theta \right)=2Q_{2}\left({\hat{G}}_{k},V\right)\alpha \left(\theta \right)+\eta e_{2}=0$$

Similarly, according to Equation (18), we get $\alpha \left(\theta \right)=\varepsilon Q_{2}^{-1}\left({\hat{G}}_{k},V\right)e_{2}$, where *ε* is a constant. For $e_{2}^{H}\alpha \left(\theta \right)=1$,$\varepsilon =\frac{1}{e_{2}^{H}Q_{2}^{-1}\left({\hat{G}}_{k},V\right)e_{2}}$, to get $\hat{\alpha }\left(\theta \right)=\frac{Q_{2}\left({\hat{G}}_{k},V\right)e_{2}}{e_{2}^{H}Q_{2}\left({\hat{G}}_{k},V\right)e_{2}}$. *V* can be estimated as:

(19) $$\hat{V}=\underset{V}{\max}e_{2}^{H}Q_{2}\left({\hat{G}}_{k},V\right)e_{2}$$

Through a 1D search, the ${\hat{V}}_{k}\left(k=1,\cdots ,\hat{K}\right)$ of the *k*-th target can be obtained, and the range of *k*-th target can be obtained from:

(20) $${\hat{R}}_{k}={\hat{G}}_{k}-\frac{f_{c}}{S}{\hat{V}}_{k}$$

Finally, using least square method to estimate the angle of *k-*th target, taking ${\hat{G}}_{k}$ and ${\hat{V}}_{k}$ into $\hat{\alpha }\left(\theta \right)$, we can get:

(21) $$\hat{\alpha }\left(\theta_{k}\right)=\frac{Q_{2}\left({\hat{G}}_{k},{\hat{V}}_{k}\right)e_{2}}{e_{2}^{H}Q_{2}\left({\hat{G}}_{k},\hat{V}\right)e_{2}}$$

for $\alpha (\theta )={\left[1,e^{j2\pi \frac{f_{c}}{c}d\sin\theta },\cdots ,e^{j2\pi \frac{f_{c}}{c}d\left(h_{1}-1\right)\sin\theta }\right]}_{1\times h_{1}}^{Τ}$, let ${\hat{q}}_{k}=phase\left[\hat{a}\left(\theta \right)\right]$, ${\hat{q}}_{k}$ can be expressed as:

(22) $$\begin{array}{l} {\hat{q}}_{k}={\left[0,\frac{f_{c}}{c}d,\cdots ,\frac{f_{c}}{c}d\left(h_{1}-1\right)\right]}^{Τ}\cdot \left(2\pi \sin\theta_{k}\right)\\ \;\;\;\;\;=p\cdot b_{k} \end{array}$$

where $p={\left[0,\frac{f_{c}}{c}d,\cdots ,\frac{f_{c}}{c}d\left(h_{1}-1\right)\right]}^{Τ}$,$b_{k}=2\pi \sin\theta_{k}$, the least square method as:

(23) $$\underset{b_{k}}{\min}{\left\|p\cdot b_{k}-{\hat{q}}_{k}\right\|}_{2}^{2}$$

The solution of *b* is $b_{k}={\left(p^{Τ}p\right)}^{-1}p^{Τ}{\hat{q}}_{k}$, the *θ* can be estimated as:

(24) $${\hat{\theta }}_{k}=\arcsin\left(\frac{b_{k}}{2\pi }\right)$$

Therefore, only $1+\hat{K}$ 1D searches are used to estimate the range, velocity, and angle of the targets, which greatly reduces the computational complexity of 3D the search.

**Figure 6:**
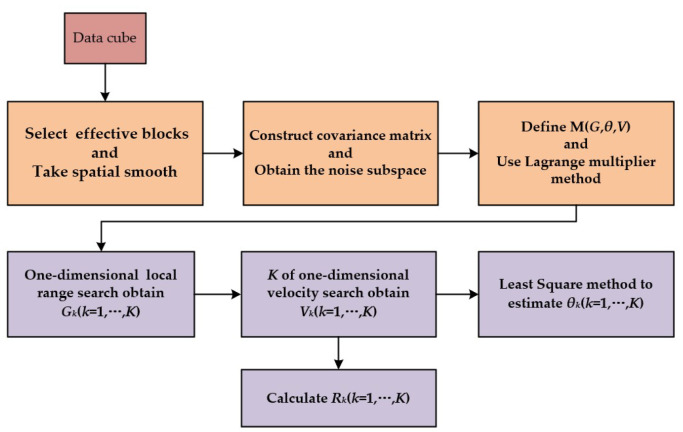
Flowchart of the proposed low complexity algorithm.

We summarize the steps for the proposed low complexity algorithm in Figure 6.

## 4. Experimental Results and Performance Analysis

This section presents the results of several experiments, mainly including three simulation experiments in Section 4.1 and four actual indoor and outdoor experiments in Section 4.2, and the performance analysis of the proposed algorithm in Section 4.3.

### 4.1. Simulation Experiment

We consider the simulated FMCW radar parameters as carrier frequency is *f_c_* = 77 GHz, the sweep duration is *T_c_* = 40 μs, the signal bandwidth is *B_w_* = 300 MHz, the time sampling frequency is *f_s_ =* 7 MHz, the number of time samples is *M* = 1/*f_s_* = 280, the number of Chirps is *L* = 12, the number of array antennas is *N* = 6, and the spacing of antenna is *d* = *λ*/2. The truncated length in the temporal domain for Block construction illustrated in Figure 3 is selected as 10, namely, *m*_2_−*m*_1_ + 1=*m*_4_−*m*_3_ + 1 = 10. The size of the spatial smoothing window is set to: *h*_1_ = 4, *h*_2_ = 250, *h*_3_ = 8, *p*_1_ = 2, *p*_2_ = 30, *p*_3_ = 4.

#### 4.1.1. Detection Simulation

**Figure 7:**
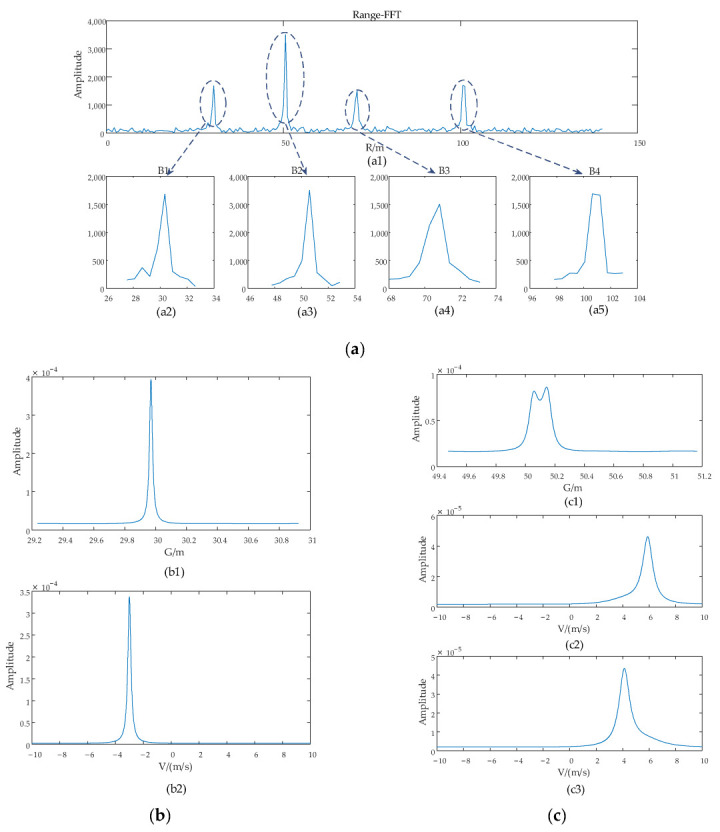

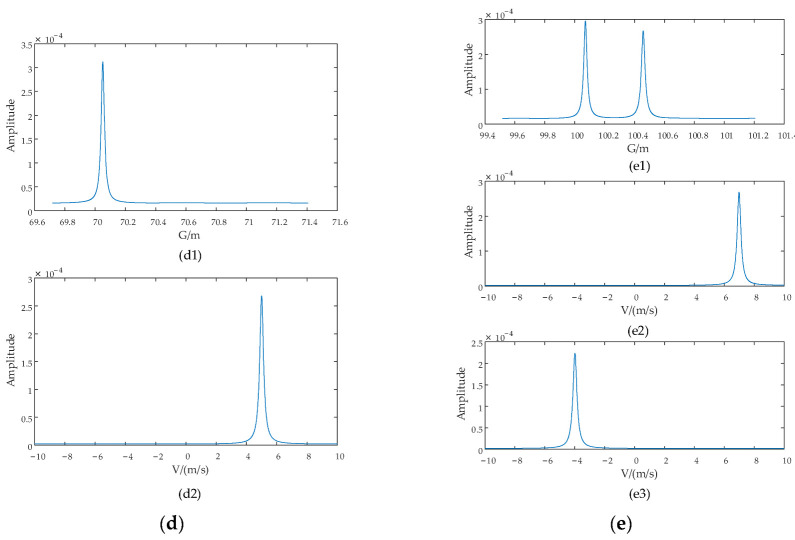
Simulation results of experiment for six targets at SNR = 10 dB. (**a**) is the targets-located blocks selection for range estimation. (**b**) is the *G* and *V* estimation process of B1. (**c**) is the *G* and *V* estimation process of B2. (**d**) is the *G* and *V* estimation process of B3. (**e**) are the *G* and *V* estimation process of B4.

The first simulation experiment is conducted for verification of effectiveness of the proposed algorithm under the scenario with six targets, Target 1 [30 m, −3 m/s, −20°], Target 2 [50 m, 4 m/s, 35°], Target 3 [50.1 m, 6 m/s, 20°], and Target 4 [70 m, 5 m/s, 40°], Target 5 [100 m, 7 m/s, −30°], Target 6 [100.5 m, −4 m/s, 30°], and the proposed algorithm is used for super-resolution estimation of the targets. It can be noticed that the Target 2 and Target 3 are very close to each other with a space of 0.1 m in the range domain, and Target 5 and Target 6 are spaced with 0.5 m. The estimation process is shown in Figure 7 and final estimated results are listed in Table 1.

According to the proposed algorithm, the range, angle, and velocity can be estimated sequentially for each target following the flowchart presented in Figure 6. As shown in Figure 7, four estimated range blocks, B1 to B4 (shown in Figure 7(a1–a4)), can be selected for all targets in the first step. Obviously, the block B2 contains the range information of Target 2 and Target 3, and the block B4 contains the range information of Target 5 and Target 6 targets. Then, the cascaded estimation processes are performed for blocks B1 to B4, as shown in Figure 7b,e, respectively. To be more specific, Figure 7b presents the estimated *G*, in Figure 7(b1), and the estimated *V* for Target 1 in Figure 7(b2). Figure 7c shows the estimation process of *G* and *V* for two targets, Target 2 and Target 3; namely: Figure 7(c1) shows the estimated *G* of the two targets, and Figure 7(c2,c3) shows the corresponding estimated *V* for the two targets, respectively. Finally, through the 1D search of estimated *G* and *V*, the value of the peaks will be utilized to calculate the *G* and velocity for each target by the Equations (17) and (19), respectively, and then the estimated *G* and velocity for each target will be utilized to calculate the corresponding range and angle by the Equations (20) and (24). The final estimated results are listed in Table 1.

This simulation experiment performs the cascaded estimation process of the proposed algorithm estimating the targets, and proves the effectiveness of the algorithm under a simulated environment. The results in Table 1 show that the proposed algorithm can accurately achieve the 3D joint estimation of the range, velocity, and angle of multi-targets.

#### 4.1.2. Algorithm Accuracy

**Figure 8:**
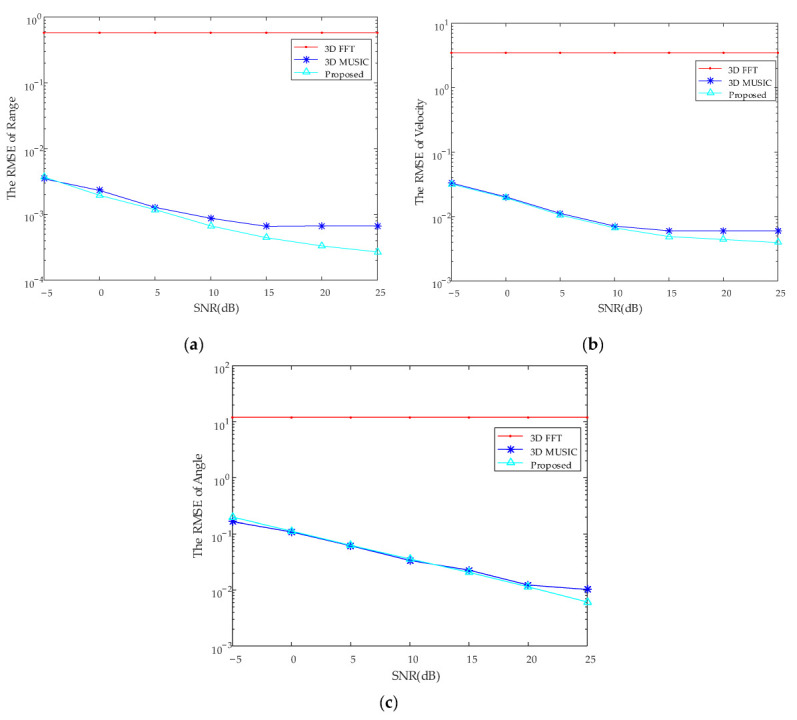
The results of accuracy comparison. (**a**) the RMSE of range. (**b**) the RMSE of velocity. (**c**) the RMSE of angle.

The second simulation experiment is to compare the accuracy of the proposed algorithm with 3D FFT and 3D MUSIC algorithm, and use root mean squared error (RMSE) to evaluate the accuracy of the algorithm. Define the RMSE as: $\frac{1}{K}\sum_{k=1}^{K}\sqrt{\frac{1}{N}{\sum_{n=1}^{N}\left(\zeta_{n}-\zeta_{k}\right)}^{2}}$, where *K* is the number of targets, *N* is the number of the experiments per target, $\zeta_{n}$ is the estimated parameters of the *N*th experiment, $\zeta_{k}$ is the parameter of set target. In this experiment, the number of detections for each target is *N* = 100 and a total of *K* = 5 targets are tested. The result is as shown in Figure 8.

Through the second simulation experiment, it can be found that the accuracy of the proposed algorithm is much higher than that of the 3D FFT algorithm under the same experimental conditions, and slightly higher than that of the 3D MUSIC algorithm. 

#### 4.1.3. Complexity Analysis

**Figure 9:**
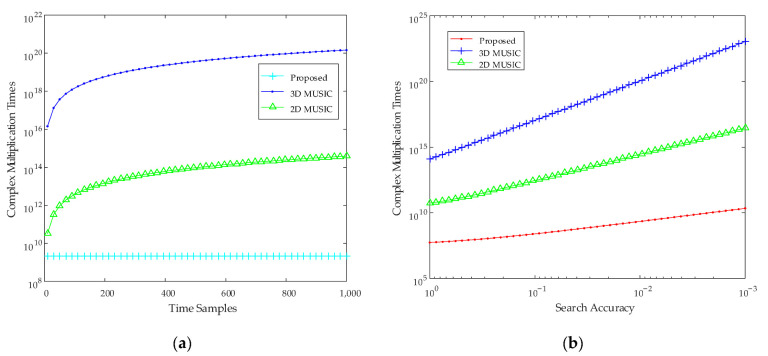
The comparison results of complexity. (**a**) the relationship between the complexity and the number of time samples when the search precision is 0.01. (**b**) the relationship between the complexity and the search precision when the number of time sample is 750.

The proposed algorithm to greatly reduce the complexity was introduced in Section 3. In this simulation experiment, we analyze its complexity and compare the complexity with 3D MUSIC and 2D MUSIC algorithm. The main contribution of complexity includes: FFT, correlation matrix, eigenvalue decomposition, and peak searching. The complexity of the proposed algorithm is *O* (*h*_1_*h*_2_*h*_3_log_2_*h*_2_ + (*mh*_1_*h*_3_)^3^ + (*h*_1_*mh*_3_ – *K*) (*n_r_h*_1_*h*_3_ (*h*_1_*h*_3_ + *m*) + *Kn_v_h*_1_ (*h*_1_ + *mh*_3_)) + *Kh*_1_). The complexity of 3D MUSIC is *O* ((*h*_1_*h*_2_*h*_3_)^3^ + (*h*_1_*h*_2_*h*_3_ – *K*) *h*_1_*h*_2_*h*_3_*n_r_n_v_n_a_*). The complexity of 2D MUSIC is *O* ((*h*_1_*h*_2_)^3^ + (*h*_1_*h*_2_ *– K*) *h*_1_*h*_2_*n_r_n_v_*), where *K* is the number of targets, *m* is the frequency domain extraction length, *n_r_* is the number of steps for range search, *n_v_* is the number of steps for velocity search, and *n_a_* is the number of steps for angle search. The result is as shown in Figure 9. The experimental results show that the complexity of the proposed algorithm is much lower than that of the 3D MUSIC algorithm, and even lower than that of the 2D MUSIC algorithm.

### 4.2. Actual Experiment

**Figure 10:**
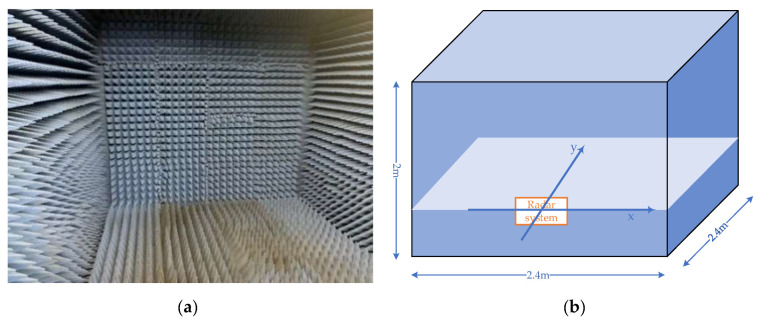
Indoor experimental environment: (**a**) the inside view of the microwave anechoic chamber; (**b**) illustration of the size of the chamber and the coordinate system.

The actual experiments will be carried out in both indoor and outdoor environments to demonstrate the feasibility of the proposed algorithm. The indoor experimental environment is in a small microwave anechoic chamber with size L × W× H 2.4 m× 2.4 m× 2 m, as shown in the Figure 10, and the outdoor experimental environment is on the road. The FMCW radar sensor system is the AWR1443 radar device of Texas Instruments (TI).

Consider the FMCW radar parameters as: the carrier frequency *f_c_ =* 77 GHz, the sweep duration *T_c_ =* 58 us, the signal bandwidth *B_w_ =* 1.16 GHz, the time sampling frequency *f_s_ =* 5 MHz, the number of time samples *M* = 1/*f_s_* = 290, the number of Chirps *L* = 12, the number of array antennas *N* = 4, the spacing of antenna *d* = *λ*/2, The length of the frequency domain extraction is 10. The size of the spatial smoothing window is set to: *h*_1_ = 3, *h*_2_ = 260, *h*_3_ = 8, *p*_1_ = 1, *p*_2_ = 30, *p*_3_ = 4.

#### 4.2.1. Corner Reflector Detection

**Figure 11:**
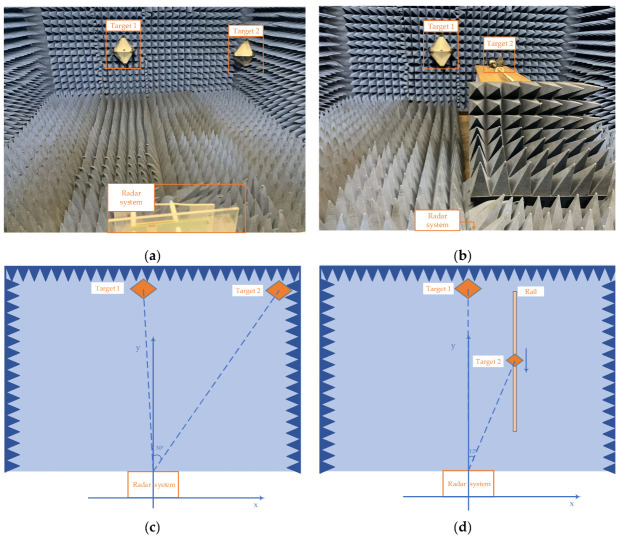
Experimental scenarios. (**a**,**c**) are the experimental scene and model diagram of experiment 1, there are two stationary targets in the experiment. (**b**,**d**) are the scene and model diagram of experiment 2, one stationary and one moving targets in the experiment.

The first and second actual experiments are detecting targets (corner reflectors with regular shape) indoors which were carried out in these experimental scenarios as shown in the Figure 11, which detected two stationary targets, one stationary and one moving targets, respectively. Specific information for each target is listed in Table 2.

**Figure 12:**
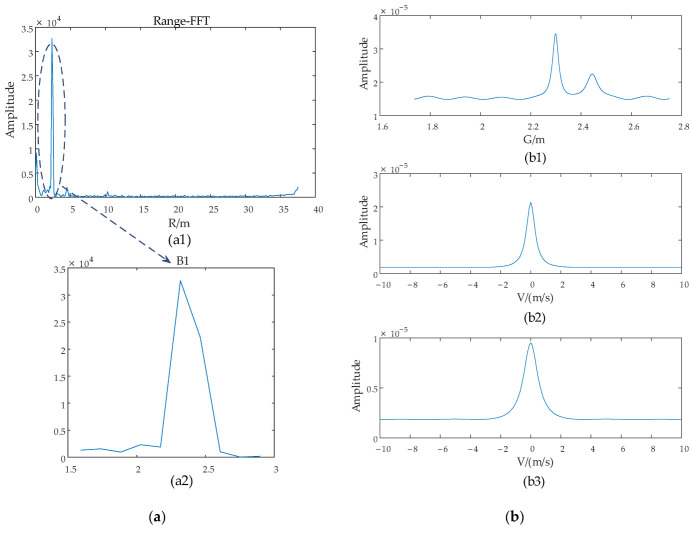

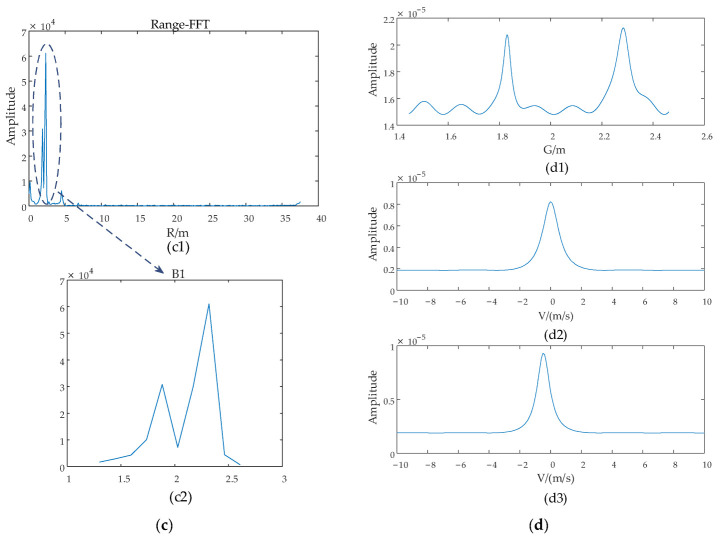
The estimation process of *G* and *V*. (**a**) is the targets-located blocks selection of experiment 1. (**b**) is the result of *G* and *V* of experiment 1. (**c**) is the targets-located blocks selection of experiment 2. (**d**) is the result of *G* and *V* of experiment 2.

After the calculation of the proposed algorithm, the estimation process of *G* and *V* is as shown in Figure 12 and final estimated results are listed in Table 3.

As with simulation experiments, the range and angle of the target can be obtained from Equations (20) and (24) after obtaining the *G* and *V*. The final estimated results are listed in Table 3.

In order to present the actual effect of the complexity-reducing effect of the proposed algorithm, the computational times of real radar data for 3D-MUSIC, 3D-FFT, and the proposed algorithm, respectively, are obtained. All implementations are performed on a dual Intel(R) Xeon(R) Gold 5218 CPU 2.3 GHz server with 128 G of memory running Ubuntu Linux 20.04, and the comparison results are listed in Table 4. The 3D-FFT is with just sub-seconds but with very low resolution. Several days are needed for the 3D-MUSIC algorithm, while the execution time of the proposed algorithm is just seconds.

The SNR of Experiment 1 and 2 is estimated as about 15 dB, and the calculated RMSEs are as shown in Table 5. The corner reflectors are selected as experimental targets, and the calculated RMSEs of range and velocity are a bit of bigger than the corresponding simulated results as shown in Figure 8a,b. The estimated angle of Target 1 of Experiment 1 is estimated as −2.8°, while the center of Target 1 (one corner reflector) is set up in the angle of −4°, and thus the RMSE of angle is around 1.49°. However, the precision of the estimated results of angle is in a very small value with about 0.028°, which is similar to the simulated RMSE result as shown in Figure 8c.

#### 4.2.2. Irregular-Shape Target Detection and 2D Imaging

**Figure 13:**
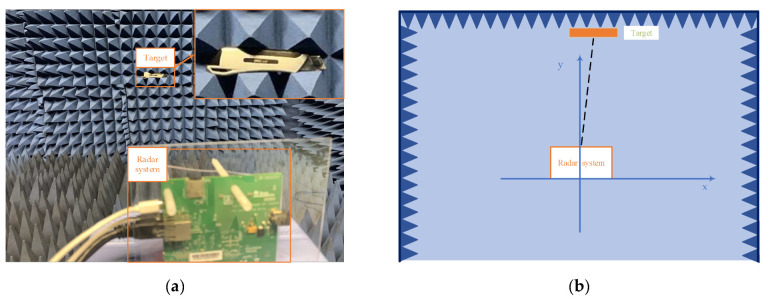
The experimental scene diagram. (**a**) is the experimental scene. (**b**) is the model diagram of experiment.

**Figure 14:**
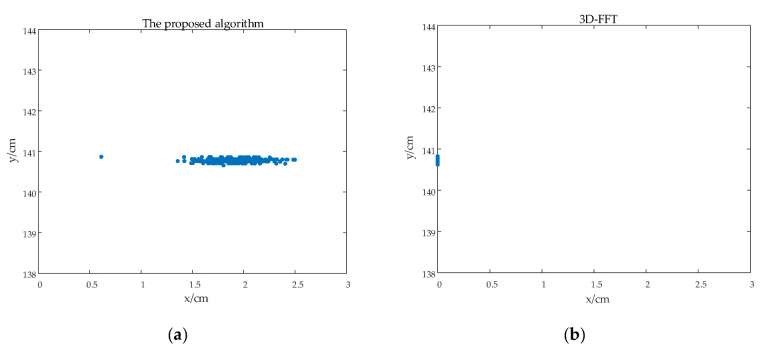
The result of the experiment. (**a**) is the imaging result of the proposed algorithm. (**b**) is the imaging result of 3D FFT algorithm.

In comparison with corner reflector detection, it is also necessary to test the proposed algorithm with irregular-shape target detection. A small metal knife with irregular shape is set up in the same plane with radar system, and the angle of its center is very mall, as shown in Figure 13a. The target is detected through 1500 trials, and then the estimated $\hat{R}$ and $\hat{\theta }$ are used to calculate the position information of $x=\hat{R}\sin\hat{\theta }\;$ and $y=\hat{R}\cos\hat{\theta }$ in the coordination system as shown in Figure 13b. As the proposed algorithm can achieve super-resolution estimation of the range, velocity, and azimuth angle of the target, a shape profile of the metal knife along the x-axis direction, namely, the projection of the target in the azimuth cross range, can be estimated successfully as shown in Figure 14a. However, 3D FFT has worse resolution, especially in the angle domain, and thus only one point-like target is estimated as shown in Figure 14b.

#### 4.2.3. Targets Detection in Outdoor Environment

**Figure 15:**
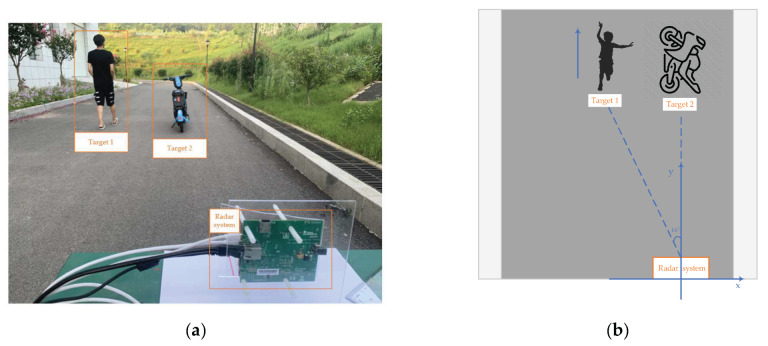
Outdoor experimental scenes. (**a**) is the outside environment. (**b**) is the model diagram of environment.

**Figure 16:**
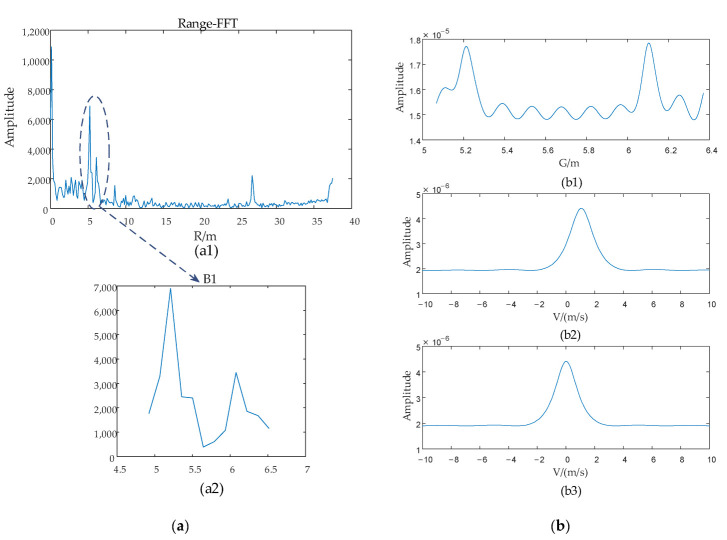
The estimation process of *G* and *V*. (**a**) is the targets-located blocks selection. (**b**) is the result of *G* and *V* of the experiment.

The fourth actual experiment is an outdoor targets detection experiment which was conducted in the scenario as shown in Figure 15. The targets of this experiment are a moving person and a stationary bicycle. The estimation process of *G* and *V* as shown in Figure 16 and the result of this experiment is listed in Table 6.

### 4.3. Discussion

A number of experimental trials were conducted in this section to verify the effectiveness of the proposed algorithm. Experimental results show that the proposed algorithm can successfully achieve the joint 3D estimation of range, velocity, and angle for multi-targets with the characteristics of super resolution and low complexity when compared with the conventional 3D FFT and 3D MUSIC algorithms. Moreover, the accuracy of the proposed algorithm is much higher than that of the 3D FFT algorithm under the same experimental conditions, and slightly higher than that of the 3D MUSIC algorithm because it slightly improves the SNR. According to the analysis in Subsection 4.1.3, the complexity of the proposed algorithm is lower than that of the conventional 3D MUSIC algorithm, and even lower than that of the 2D MUSIC algorithm. However, similarly to the conventional 3D MUSIC, the proposed method is still composed of a variety of matrix operations, such as EVD, and two operations of 1D spectrum searching are inevitable. Thus, it is still difficult to implement the low complexity algorithm on FPGA and DSP for real time application.

## 5. Conclusions

This paper has presented a low complexity 3D joint super-resolution estimation algorithm for an FMCW radar system, implemented by the Lagrange multiplier method and rank-reduced techniques. Various experiments, including simulation experiments, corner reflector detection and irregular-shape target detection in the chamber, and the real person and bike detection in outdoor environment, were conducted to clarify the proposed algorithm. The experimental results verified the super resolution and low complexity performance. However, it was necessary to operate the proposed algorithm on a PC due to the amount of matrix operations and searching costs. Development of a simpler version of the suggested algorithm and novel hardware design would be needed for a real-time detection FMCW radar system.

## Figures and Tables

**Table 1:** The estimated results of the first experiment.

Target Number	$\hat{R}$	$\hat{V}$	$\hat{\theta }$
1	29.9976 m	−2.9965 m/s	−20.1844°
2	50.0114 m	4.087 m/s	34.2881°
3	50.0857 m	5.8879 m/s	20.7431°
4	69.9981 m	5.0175 m/s	40.2631°
5	100.0015 m	6.9885 m/s	−29.9204°
6	100.5007 m	−3.987 m/s	29.9461°

**Table 2:** Specific information of targets.

	Target 1	Target 2
Experiment 1	[2.312 m, 0 m/s, −4°]	[2.403 m, 0 m/s, 30°]
Experiment 2	[2.295 m, 0 m/s, 0°]	[1.832 m, −0.5 m/s, 12°]

**Table 3:** The estimated results of experiment 1 and experiment 2.

	Target No.	$\hat{R}$	$\hat{V}$	$\hat{\theta }$
Experiment 1	1	2.2987 m	0 m/s	−2.8483°
	2	2.4447 m	0 m/s	28.1479°
Experiment 2	1	2.2834 m	0 m/s	−0.26748°
	2	1.8321 m	−0.5 m/s	11.7486°

**Table 4:** The comparison results of computational times for real radar data.

	3D-FFT	3D-MUSIC	The Proposed Algorithm
Experiment 1	0.1115 s	3days	1.4936 s
Experiment 2	0.1123 s	3days	1.5018 s

**Table 5:** The calculated RMSE for actual experiments.

	*R*	*V*	*θ*
Experiment 1	0.0273 m	0 m/s	1.4913°
Experiment 2	0.0721 m	0.0630 m/s	0.8776°

**Table 6:** The estimated results of experiment 4.

	Target No.	$\hat{R}$	$\hat{V}$	$\hat{\theta }$
Experiment 4	1	6.0998 m	1.1 m/s	−16.3641°
	2	5.2129 m	0 m/s	−2.1378°
